# Micronutrients in Autoimmune Diseases: Shining a Light on Vitamin D, Cobalamin, Folate, and Iron Metabolism

**DOI:** 10.3390/nu18040561

**Published:** 2026-02-08

**Authors:** Paola Triggianese, Giuseppe A. Ramirez, Francesca Cedola, Stefania Nicola, Giulia Costanzo, Luisa Brussino, Francesca Chiereghin, Davide Firinu, David Della-Morte, Vincenzo Patella, Cinzia Milito

**Affiliations:** 1 Department of Biomedicine and Prevention, Nutrition and Geriatrics, Tor Vergata University Hospital, 00133 Rome, Italy; paola.triggianese@gmail.com (P.T.); david.dellamorte@uniroma2.it (D.D.-M.); 2 Unit of Immunology, Rheumatology, Allergy and Rare Diseases, IRCCS Ospedale San Raffaele, 20132 Milan, Italy; 3 Faculty of Medicine, Università Vita-Salute San Raffaele, 20123 Milan, Italy; 4 Immunology and Allergy Unit, Department of Medical Sciences, Mauriziano Hospital, University of Turin, 10124 Turin, Italy; 5 Department of Public Health and Medical Science, University of Cagliari, 09124 Cagliari, Italy; giuliacostanzo14@gmail.com (G.C.); davide.firinu@unica.it (D.F.); 6 Department of Internal Medicine, “Santa Maria della Speranza” Hospital, ASL Salerno, 84131 Salerno, Italy; 7 Postgraduate Program in Allergy and Clinical Immunology, University of Naples “Federico II”, 80131 Naples, Italy; 8 Department of Molecular Medicine, “Sapienza” University of Rome, 00161 Rome, Italy; cinzia.milito@uniroma1.it

**Keywords:** autoimmunity, cobalamin, folate, immune system, iron, micronutrients, nutrition, vitamin D

## Abstract

**Background**: Autoimmune diseases (AIDs) are characterized by chronic inflammation and tissue damage resulting from abnormal immune responses. While genetic and environmental factors play significant roles in disease development, essential micronutrient deficiencies (MNDs) represent a critical and often overlooked contributor. **Methods**: This review examines the interactions between micronutrients and immune cells, focusing on vitamin D, vitamin B12, folate (FA), and iron, and their roles in AIDs, such as rheumatoid arthritis, autoimmune thyroid disorders, multiple sclerosis, systemic lupus erythematosus, and other connective tissue diseases. We explore the immunomodulatory effects of these micronutrients, their impact on immune tolerance, and the mechanisms by which MNDs contribute to disease progression. **Results**: MNDs are commonly observed in patients with AIDs and are associated with worsening immune dysregulation, increased inflammation, and disease severity. Vitamin D plays a pivotal role in modulating immune responses and attenuating inflammation, while iron and FA are essential for immune cell proliferation and function. Vitamin B12 supports methylation processes and genomic stability. **Conclusions**: MNDs significantly influence the pathogenesis and progression of AIDs. Routine micronutrient screening and targeted supplementation should be considered as part of clinical management, offering potential adjunctive benefits alongside conventional therapies. Further research is needed to define optimal dosing strategies and to identify patient subgroups most likely to benefit from nutrition-based interventions.

## 1. Introduction: Micronutrient Deficiencies as Critical Factors in Autoimmune Diseases

Autoimmune diseases (AIDs), which impact approximately 7–10% of the worldwide population, represent a heterogeneous group of chronic conditions characterized by inappropriate immune reactions against self-antigens, resulting, over time, in progressive tissue damage and persistent inflammation [1,2]. These disorders include both systemic conditions, such as systemic lupus erythematosus (SLE), systemic sclerosis (SSc), rheumatoid arthritis (RA), and organ-specific diseases, such as type 1 diabetes (T1D) and multiple sclerosis (MS) [1]. In this context, spondyloarthritis (SpA) and inflammatory bowel diseases (IBD) can be classified at the crossroads between AIDs and autoinflammatory disorders. Although genetic contribution, environmental exposures, and dysbiosis contribute to disease susceptibility, a critical and often underestimated factor is the status of essential micronutrients [3].

Micronutrients, comprising essential vitamins and minerals, are fundamental to human physiology. They sustain the synthesis of enzymes, hormones, and other biomolecules indispensable for tissue maintenance, metabolic balance, and overall homeostasis [4]. Micronutrients include trace elements showing absorption rates in the range of 1 to 100 mg/day. Examples of these moieties encompass iron, zinc, copper, fluoride, iodine, and manganese. Vitamin E and vitamin B6 are also usually absorbed at the same rates as trace elements. Rarer nutrients with absorption rates < 1 mg/day, such as boron, selenium, chromium, vanadium, molybdenum, and arsenate, are conventionally classified as ultra-trace elements. Folic acid (FA), vitamin D, cobalamin (B12), and vitamin A are also absorbed at comparable rates. Minerals such as sodium, potassium, calcium, magnesium, phosphorus, and chlorine are absorbed at a rate of >100 mg/day and are usually termed “macroelements”. The absorption of vitamin C shows a similar order of magnitude. Despite their minimal quantitative requirements, micronutrients are essential for preserving cellular integrity and systemic equilibrium. These compounds act as cofactors, gene expression regulators, and modulators of oxidative and metabolic pathways. The crucial role of micronutrients is underscored by the fact that even mild deficiencies or imbalances may result in severe consequences for immunity, growth, neurocognitive function, and metabolic health [4,5].

In addition to their metabolic roles, micronutrients exert significant immunomodulatory effects, acting both on innate and adaptive immune responses. They regulate macrophage and lymphocyte activity, antibody production, and the imbalance between pro- and anti-inflammatory cytokines [1,3,6]. Consequently, micronutrient deficiencies constitute a significant, yet frequently underrecognized, component of autoimmune disease management.

Circulating micronutrient concentrations are shaped by a wide array of determinants, including dietary patterns, intestinal microbiota, age, gender, and genetic background. Across AIDs, these deficiencies may also be driven by a combination of impaired gastrointestinal absorption, chronic inflammation, and autoimmune-mediated tissue damage [6,7]. Deficits in B12, vitamin D, FA, and iron are frequently reported in AIDs and may contribute to anemia, neurocognitive dysfunction, bone fragility, and altered immune regulation. The interplay between micronutrient levels and immune tolerance is complex and mediated through various cellular and biochemical pathways. Vitamin D has immunomodulatory properties and takes part in the formation of a tolerogenic milieu affecting both innate and adaptive immune responses [8]. B vitamins, particularly B12 and FA, are central to one-carbon metabolism, a network of reactions crucial to DNA synthesis, amino acid homeostasis, antioxidant generation, and epigenetic regulation [9]. Iron is required for erythropoiesis, mitochondrial respiration, redox control, and enzyme activities in immune cells [10]. Consequently, micronutrient deficiencies (MNDs) can trigger or maintain a vicious cycle of immune dysregulation–inflammation, leading, over time, to nutrient depletion. The recurrence of multiple MNDs across diverse autoimmune conditions highlights the need to incorporate nutritional assessment into routine immunological workups [1].

Understanding these mechanisms is essential for the development of tailored nutritional interventions aimed at restoring immune homeostasis and mitigating the progression of autoimmune damage [11].

## 2. Interaction Between Micronutrients and Immune Cells

**Figure 1:**
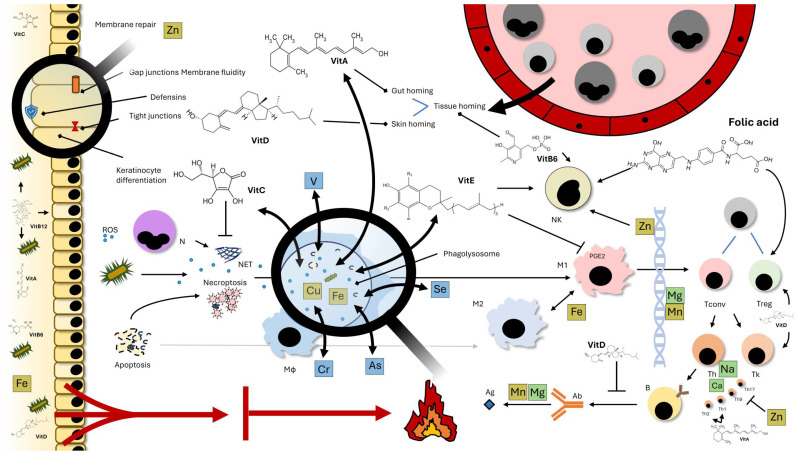
Micronutrients, including trace elements (yellow boxes) and ultra-trace elements (blue boxes), along with other elemental moieties (green boxes), are involved in multiple crucial aspects of immunity, such as steady-state immune surveillance, acute-phase responses, and chronic maladaptive inflammation. Mucosal barrier stability depends on efficient repair and renewal responses, which, in turn, rely on the availability of nutrients, such as zinc or vitamin C. Trimming of mucosal permeability to exogenous moieties, including microorganisms, toxins, and nutrients, is crucial to prevent unsolicited inflammation and facilitated triggering of autoimmunity. In this setting, the expression of defensins and mediators of intercellular communication might be modulated by vitamin D and vitamin A, with the latter also affecting membrane fluidity. Variations in the microbiome also modulate the bioavailability of nutrients such as B12. Inflammation can affect the efficiency of absorption of some micronutrients, such as iron, hindering microbial proliferation but also impairing defensive functions, including the generation of oxidative stress by phagocytes and even regulatory anti- inflammatory responses. By affecting the properties of interface tissues, micronutrients are also involved in leukocyte extravasation (vitamin B6), along with skin (vitamin D) or gut (vitamin A) homing. Phagocytosis and the generation of reactive oxygen species (ROS) to perform the oxidative burst play a crucial role in pathogen and cell death debris neutralization and disposal. In this setting, buffering of ROS is also crucial to prevent tissue damage and uncontrolled inflammation. These functions depend on highly reactive elements such as iron or moieties with intrinsic bactericidal properties such as copper. Rarer elements such as selenium, chromium, arsenate, and maybe vanadium have been hypothesized to potentially substitute at least part of the iron/copper function. Iron has also been shown to participate in the shift between pro-inflammatory (M1) and anti-inflammatory (M2) macrophages. Dampening of M1 functions has also been attributed to vitamin E. Vitamin C can act upstream by reducing the rates of formation of neutrophil extracellular traps (NET), a major source of autoantigens along with necroptotic debris, potentially fueling autoimmunity. DNA plasticity and effective access to DNA are crucial for the expansion and maturation of immune cells. In this setting, Zn-dependent enzymes, along with Mg, are required for chromatin physiology. Folic acid and vitamin B6 are also crucial to ensure effective cell replication. Other micronutrients contribute to modulating T- and B-lymphocyte differentiation and function. Vitamin D has an important role in favoring T-regulatory responses and dampening antibody-mediated responses, while sodium overload or deprivation and downstream alterations in intracellular calcium promote conventional T-cell differentiation and pro-inflammatory responses. Zn and vitamin A affect T-helper cell polarization, with Zn inhibiting Th19/Th17 differentiation and vitamin A promoting both Th1- and Th2-biased responses. Magnesium concurs with efficient antibody–antigen interactions. In this and other functions, a potential backup role has been proposed for manganese.

The immune system comprises a wide range of coordinated processes mediated by both humoral and cellular components, integrating nearly every organ and tissue in a highly dynamic manner [12]. These characteristics are finely regulated and intersect with other fundamental biological processes such as nutrient metabolism, neuroendocrine functions, and host-microbiome interactions, which, in turn, have a bidirectional influence on the bioavailability of micronutrients and their modulatory roles in inflammation [13,14]. Within this integrated framework, micronutrients modulate key processes of immune activation, including antigen presentation, cytokine secretion, lymphocyte differentiation, and the resolution of inflammation [15,16] (Figure 1). Conversely, micronutrient deficiencies are frequently observed in AIDs and arise from multiple factors, primarily impaired gastrointestinal absorption, chronic inflammation, or direct autoimmune-mediated tissue injury. As a result, MNDs may amplify autoreactive responses and reduce the efficiency of regulatory mechanisms that ordinarily maintain immune tolerance.

Dynamic interactions with the surrounding environment are crucial for the host’s integrity. Maintenance of functional interfaces, able to avoid exogenous *noxae* while facilitating nutrient absorption, constitutes a basic function in the context of innate immunity. Aberrations in barrier integrity and selectivity have instead been implicated in a wide range of immune-mediated disorders. Cell junctions such as tight junctions and gap junctions (along with desmosomes) play a fundamental role in defining the properties of epithelia and other tissues, especially in terms of fluidity and permeability. Vitamin A and other retinoids affect tight junction and gap junction expression, which in turn can have a modulatory role in immune-mediated disorders. Their role is particularly relevant in gut homeostasis, where retinoids also contribute to epithelial integrity and appropriate homing of T and B cells in the intestinal tract.

Phagocytosis, clearance of pathogens, and other potential causes of damage and/or uncontrolled inflammation are crucial tasks in the deployment of innate immune responses. Micronutrients with intrinsic chemical reactivity and/or microbicidal properties, such as iron or copper, play a fundamental role in this setting. At least part of their catalytic functions is thought to be possibly substituted by other rarer elements, including selenium, chromium, arsenate, and (with far less definite evidence) vanadium.

Effector functions of the adaptive immune response rely on accurate antigen recognition and binding. Micronutrients are involved in this process: elements such as magnesium are, in fact, required for efficient antibody responses. Manganese is thought to be a potential backup for magnesium in this setting, in line with a model of functional robustness ensured by redundancy.

Plasticity and quick responsiveness constitute additional cornerstone features of the immune response. Their biological correlations at a cellular and molecular level are readiness for cell proliferation, targeted gene editing (e.g., for T- and B-cell receptor maturation), and functional specialization following environmental stimuli. Multiple trace elements are involved in chromatin plasticity and cell differentiation within and beyond the immune system. Most robust evidence in this regard has been acquired for zinc (especially as a key component of multiple enzymes) and magnesium. Along with more complex nutrients such as vitamin A and D, zinc also contributes to shaping immune cell differentiation (Figure 1).

## 3. Role of Vitamin D in AIDs

**Figure 2:**
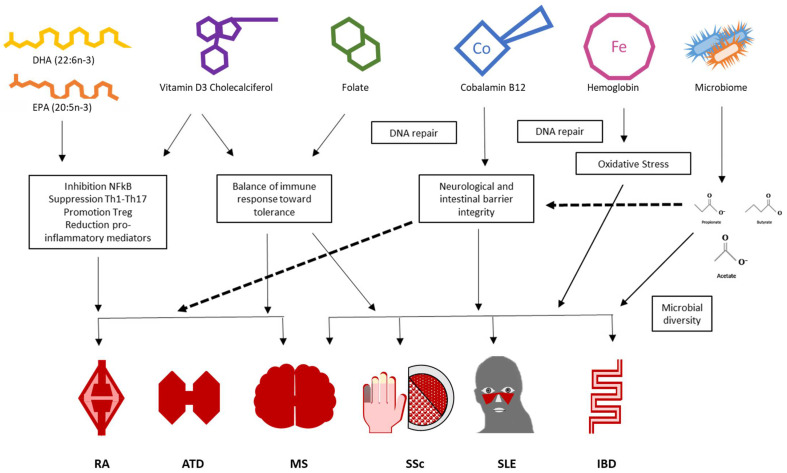
Micronutrients and autoimmune diseases: the main mechanisms. ATD: autoimmune thyroid disorders; DHA: docosahexaenoic acid; EPA: eicosapentaenoic acid; IBD: inflammatory bowel diseases; MS: multiple sclerosis; RA: rheumatoid arthritis; SLE: systemic lupus erythematosus; SSc: systemic sclerosis.

Vitamin D, a fat-soluble secosteroid, is increasingly recognized as a key immunomodulatory micronutrient influencing the onset and course of AIDs (Figure 2). Beyond its long-recognized endocrine role in calcium and phosphate homeostasis, its active metabolite, 1,25-dihydroxyvitamin D3 (calcitriol), exerts biological activity through the vitamin D receptor (VDR), expressed in numerous immune cells, including dendritic cells, macrophages, T lymphocytes, and B cells [12].

The binding of calcitriol to VDR regulates the transcription of multiple genes containing vitamin D response elements, thereby influencing both innate and adaptive immune responses. Mechanistically, vitamin D suppresses dendritic cell maturation and antigen presentation, reduces the production of pro-inflammatory cytokines (interleukin (IL)-2, IL-17, interferon (IFN)-γ, tumor necrosis factor (TNF)-α), and enhances the secretion of anti-inflammatory cytokines such as IL-10 [13]. It promotes the differentiation of regulatory T cells (Treg) while inhibiting the proliferation and the antibody production of B lymphocytes, ultimately contributing to the maintenance of immune tolerance [14] (Figure 1 and Figure 2). Moreover, calcitriol inhibits the NF-kB signaling pathway and induces the expression of antimicrobial peptides (cathelicidin and beta-defensin 2), thus reinforcing mucosal immune defense mechanisms [15,16].

Vitamin D deficiency has consistently been associated with a higher risk of developing tissue/organ-limited autoimmune conditions such as autoimmune thyroid disorders (ATD) and MS, along with systemic disorders such as SLE, RA, psoriasis, and inflammatory bowel diseases (IBD). Lower vitamin D levels have also been associated with more severe disease courses in these settings [17,18]. In ATD, the 25(OH)D levels are significantly lower in Hashimoto’s thyroiditis and Graves’ disease patients than in healthy controls, and vitamin D deficiency is common in both overt and subclinical disease [19,20].

Several studies have reported an inverse correlation between 25(OH)D levels and anti-thyroid peroxidase (anti-TPO) and anti-thyroglobulin antibody titers, as well as with thyroid-stimulating hormone (TSH) levels, suggesting that lower vitamin D is associated with higher autoimmune activity [21,22,23]. Supplementation with vitamin D in vitamin D-deficient, euthyroid TPO antibody-positive individuals has been associated with reductions in anti-TPO titers, although effects appear less pronounced in established hypothyroidism [24,25,26].

In T1D and MS, adequate vitamin D levels during early life appear to be protective against islet and myelin autoimmunity, respectively; however, supplementation after disease onset has produced inconsistent outcomes [27,28,29]. In MS patients, vitamin D deficiency and insufficiency are associated with increased risk of disease onset and higher relapse rates, supporting a potential role for vitamin D in both susceptibility and disease modulation [30,31].

Similar patterns are observed in SLE. In SLE, vitamin D deficiency is frequent, and lower 25(OH) vitamin D levels are consistently associated with higher disease activity scores and, in some cohorts, with organ involvement such as lupus nephritis [32,33,34]. A study involving 60 SLE patients observed that those with active disease had significantly lower serum vitamin D compared to the inactive disease group, with a moderate inverse correlation between activity scores and vitamin D concentrations [35]. Juvenile-onset SLE patients also show reduced 25(OH)D concentrations compared to controls with inverse correlations between vitamin D, disease activity indices, and IFN-γ concentrations, consistent with a role for vitamin D in modulating inflammatory cytokine networks [36].

In RA, mean vitamin D levels tend to be lower than in the general population, and some studies report weak, but negative correlations, between vitamin D and disease activity [33,37,38].

Seronegative SpA constitutes a heterogeneous set of immune-mediated disorders lying pathogenically at the crossroads between autoinflammation and autoimmunity [39,40,41,42]. Within the large spectrum of clinical and pathophysiological manifestations characterizing SpA, besides axial skeleton inflammation, dysregulation of intestinal homeostasis is thought to play a crucial role and potentially account for the frequent overlap with IBD [43,44] and for the disproportionately high prevalence of MNDs, including vitamin D in this group of diseases [45,46] (Figure 2). In patients with SpA, vitamin D deficit has been correlated with radiographic sacroiliitis and disease severity [46,47].

Even more intriguing data about potential clinical/pathophysiological association with vitamin D deficits have been acquired in the setting of SSc. In fact, besides generic evidence of low vitamin D levels in patients with SSc (like other immune-mediated disorders), mechanistic studies suggest a potential involvement of vitamin D in modulating the disease-defining fibrotic events causing irreversible tissue/organ damage. Furthermore, the physiological mechanism that implicates vitamin D in calcium homeostasis might be subverted in SSc to promote soft tissue calcinosis [48].

Although the causal relationship between vitamin D status and autoimmunity remains incompletely defined, converging mechanistic and clinical data support its role as a critical immunoregulatory micronutrient that promotes self-tolerance and restrains chronic inflammation and disease activity [49]. For these reasons, vitamin D supplementation, a safe and low-cost adjunct to conventional therapies, may contribute to attenuating autoimmune inflammation and restoring immune homeostasis. Future studies are warranted to clarify optimal dosing strategies, long-term effects, and the impact of VDR genetic polymorphisms on individual responses.

## 4. FA and Iron: Interconnected Micronutrient Dynamics in AIDs

FA (vitamin B9) and iron represent two tightly interconnected micronutrients whose deficiencies are common in AID patients and exert effects on vascular biology, hematopoiesis, and immune regulation (Figure 1 and Figure 2). FA is a water-soluble vitamin, indispensable for nucleotide synthesis, amino acid metabolism, and one-carbon transfer reactions, cooperating with B12 to regulate homocysteine remethylation and S-adenosyl methionine (SAM) methylation [9,50]. Iron is essential for erythropoiesis, oxidative metabolism, and a variety of enzyme systems, including those involved in innate and adaptive immune response. Deficiency in either can lead to deleterious effects, contributing to anemia, fatigue, and impaired immune function [51].

In autoimmune endocrine settings, such as ATD, both FA and iron deficiencies have been described with increased frequency. Subclinical hypothyroidism has been associated with high prevalence of iron deficiency anemia and a strong inverse correlation between TSH and hemoglobin levels, suggesting that both overt and subtle thyroid dysfunction can coexist with iron deficiency [52]. In T1D, the presence of parietal cell antibodies has been linked to higher rates of anemia and reduced ferritin, indicating that gastric autoimmunity can compromise both B12 and iron status even in non-gastrointestinal AIDs [53,54].

Iron deficiency is also highly reported across rheumatic diseases and frequently contributes to anemia, which affects approximately 35 to 50% RA and SLE patients [54]. Patients with SSc also show high rates of iron-deficient anemia, especially in the case of concomitant pulmonary arterial hypertension [55,56,57]. Iron-deficient anemia constitutes a complicating/precipitating factor for respiratory insufficiency in patients with pulmonary hypertension and might share some pathogenic mechanisms, such as alterations in IL6 balance [57].

In SpA, anemia and low ferritin levels occur predominantly in women and are strongly linked with higher disease activity and impaired function [58,59]. Pro-inflammatory cytokines, particularly IL-6 and TNF-α, upregulate hepatic hepcidin, thereby promoting ferroportin degradation and reducing intestinal iron absorption [60,61]. This leads to intracellular iron sequestration and functional iron deficiency, which often coexists with iron depletion in chronic inflammatory states [62,63] (Figure 1).

FA deficiency is relatively common in AIDs and is frequently related to malabsorption or increased metabolic demand. In SpA, FA deficiency affects about one-fifth of patients and often overlaps with B12 low levels [17,18]. Since FA is essential for processes modulating immune responses and inflammatory gene expression (like one-carbon metabolism, nucleotide synthesis, and methylation reactions), its depletion can therefore contribute to elevated homocysteine, increased inflammatory stress, and impaired methylation capacity. Lower FA levels are particularly evident in patients with coexisting IBD [17,18]. Methotrexate (MTX), an antimetabolite with immunosuppressive and antineoplastic properties, used as a cornerstone disease-modifying antirheumatic drug (DMARD) in AIDs, including SLE and RA, inhibits dihydrofolate reductase, disrupting folic acid metabolism, and interferes with DNA synthesis and repair and with cellular replication. Between 7% and 30% of people receiving MTX discontinue it in the first year due to adverse effects that are likely due to FA antagonism. The most common adverse effects include mouth ulcers, gastrointestinal disturbance, bone marrow toxicity, and hepatic toxicity, which might be prevented with FA supplementation. Based on this concern, adding FA in patients on MTX is essential to reduce side effects. Generally, this occurs on a day other than the one when MTX treatment is administered. FA supplementation is continued as long as MTX is prescribed [64].

In MS, iron accumulates abnormally in the central nervous system and may contribute to disease progression. This iron accumulation can amplify microglial activation and promote oxidative stress through reactive oxygen species formation, potentially contributing to neurodegeneration [65].

Consequently, iron deficiency represents a frequent and clinically relevant micronutrient disturbance in AIDs and is a major contributor to their characteristic anemia patterns. This deficiency, arising from a combination of chronic inflammation, autoimmune-mediated tissue damage, and impaired gastrointestinal absorption, contributes to clinical symptoms and reduces immune competence, compounding the overall disease burden. Recognizing and addressing iron deficiency in autoimmune patients is therefore crucial, as targeted supplementation or correction of underlying inflammatory mechanisms can improve outcomes and quality of life.

## 5. B12 Deficiency in AIDs

B12, obtained exclusively from animal-derived food, is essential for genomic stability and immune cell function through its role in homocysteine remethylation, nucleotide synthesis, and methylation processes; this makes adequate B12 levels essential for maintaining balanced T-cell subsets and a physiological CD4/CD8 ratio [66]. Its deficiency is common across several AIDs and impairs lymphocyte proliferation and antioxidant defenses, potentially amplifying inflammation and immune dysregulation [67,68] (Figure 1 and Figure 2).

In ATD, including Hashimoto’s thyroiditis and Graves’ disease, borderline or low B12 levels are commonly observed. A study involving 133 hypothyroid patients showed that borderline-to-low B12 levels were more frequent in both overt and subclinical hypothyroidism groups compared with controls, suggesting a subclinical B12 vulnerability in this population [69]. Another case–control study on subclinical hypothyroid patients reported a non-significantly higher level of B12 among these patients compared to healthy controls, whereas holotranscobalamin (holoTC), the metabolically active fraction of B12, was significantly higher in subclinical hypothyroid patients [70]. Positive anti-TPO antibodies and severity of thyroid insufficiency are correlated with worse B12 deficiency in subclinical hypothyroid patients, suggesting a complex interplay between thyroid autoimmunity and B12 status [70].

Mechanistically, B12 serves as a cofactor in two reactions: the conversion of methylmalonyl-CoA to succinyl-CoA and the remethylation of homocysteine to methionine, the latter linking B12 to folate metabolism and SAM [71]. Deficiency, therefore, affects DNA synthesis, myelin status, methylation reactions critical for immune cell development and epigenetic programming and can lead to severe neurological deficits [72].

In fact, MS patients demonstrate elevated homocysteine levels and suboptimal B12 and FA concentrations. A meta-analysis of 21 studies, involving 1738 MS patients, documented significantly higher serum homocysteine levels compared to controls, particularly in relapsing–remitting MS [73]. In children with MS, at onset, elevated homocysteine occurs without laboratory signs of B12 deficiency, suggesting functional disruption of folate metabolism pathways [74].

Deficits in B12 are also common in rheumatic diseases, with decreased serum concentrations detected in approximately 20–25% of patients with RA and SLE [55]. However, in Segal’s study, only 26% of individuals with low serum B12 showed abnormal homocysteine or methylanomic acid levels and responded to B12 supplementation, underlining that routine serum levels may overestimate deficiency prevalence. Deficiency risk factors include vegetarian diets and potential malabsorption due to autoimmune mechanisms or medication use, such as H2 blockers or proton pump inhibitors [55].

Altogether, this evidence illustrates how B12 deficiency disrupts lymphocyte homeostasis, methylation capacity, and neuroimmune integrity. Its correction represents a low-risk, mechanistically grounded strategy that may support immune regulation and counteract inflammatory amplification in susceptible autoimmune populations [75].

## 6. Nutritional Intervention: Tracing Novel Targets

The emerging role of nutritional intervention in AIDs has gained substantial scientific attention as evidence increasingly highlights diet as a key regulator of immune homeostasis, inflammatory signaling, and disease progression. AIDs are characterized by chronic activation of the immune system and loss of self-tolerance, processes strongly influenced by metabolic and environmental factors, including nutrition. The “Western diet,” characterized by high caloric intake, processed foods, and sugar, has been identified as a driver of autoimmunity through the promotion of dysbiosis and intestinal permeability (often termed “leaky gut”), which facilitates immune hyper-activation via molecular mimicry [76,77] (Figure 1). Conversely, anti-inflammatory dietary models, such as the Mediterranean and plant-based diets, have shown to downregulate pro-inflammatory cytokines, improve endothelial function, and reduce disease activity and autoimmune inflammatory processes in conditions such as RA, SLE, and MS, reducing oxidative stress and inflammatory biomarkers [78]. Moreover, the Mediterranean diet influences immune system function and gut microbiota composition, exerting antioxidant and immunomodulatory effects in patients affected with AIDs differently from a high-energy diet, rich in animal fat and proteins, salt, and refined sugars [79].

Beyond dietary patterns, specific nutrients have emerged as immunomodulatory agents: omega-3 polyunsaturated fatty acids have been shown to inhibit NF-κB signaling and Th1 and Th17 cell responses while promoting Treg activity, contributing to immune tolerance, thereby competing with arachidonic acid to dampen the production of pro-inflammatory eicosanoids, offering symptomatic relief and reducing relapse rates across multiple autoimmune conditions [80,81,82,83] (Figure 2).

Micronutrients, including vitamin D, folate, and iron, have also been extensively studied, with growing evidence of their regulatory effects on antigen presentation, cytokine balance, and oxidative stress, with deficiencies consistently associated with increased autoimmune risk and severity through their effects on antigen-presenting cells and T-cell differentiation [84]. FA plays a pivotal role in one-carbon metabolism and DNA synthesis, and its deficiency has been associated with impaired regulatory T-cell function and heightened inflammatory responses [85]. B12 is essential for methylation reactions and neurological integrity, and low serum B12 levels are frequently reported in AIDs, where supplementation has been linked to reduced homocysteine levels and potential modulation of neuroinflammatory pathways [86]. Iron homeostasis is also increasingly recognized as an immunomodulatory factor, as both iron deficiency and overload can disrupt innate and adaptive immune responses. In AIDs such as SLE, iron supplementation has been shown to improve anemia and fatigue while indirectly influencing immune competence and inflammatory status [87].

Furthermore, novel evidence also supports the role of the gut microbiota and microbiome as a central mechanistic link between nutrition and autoimmunity. Dietary fiber, prebiotics, probiotics, and postbiotics influence microbial diversity and enhance SCFA production, which plays a critical role in maintaining intestinal barrier integrity and immune homeostasis [88]. Emerging evidence, thus, supports the use of specific bioactive compounds such as SCFA-enhancing fibers and targeted probiotic strains (e.g., *Lactobacillus* and *Bifidobacterium*) to promote regulatory T-cell differentiation and suppress pro-inflammatory Th17 responses, mechanisms relevant to several autoimmune diseases like MS, RA and IBD (Figure 1 and Figure 2) [89,90,91]. Based on current evidence, several investigational studies are ongoing to define how diet could affect AIDs in order to explore boundaries for nutritional intervention in patients with chronic rheumatic disorders (Table 1).

Additionally, novel interest has emerged in plant-derived immunomodulatory compounds, including flavonoids, curcumin, and sulforaphane, which have demonstrated epigenetic and antioxidant effects that may suppress autoimmune inflammation [92].

Novel nutritional approaches, including intermittent fasting and fasting-mimicking diets, have shown promise in reprogramming immunometabolism, reducing oxidative stress, and attenuating autoimmune pathology in both experimental models and early clinical trials [93].

Collectively, these findings highlight nutrition as a dynamic and mechanistically relevant therapeutic avenue, supporting its integration into personalized and adjunctive strategies for AID prevention and management.

## 7. Conclusions

In recent years, several studies have highlighted that essential micronutrients play a pivotal and often underestimated role in AIDs. Beyond their classical metabolic functions, vitamin D, B12, FA, and iron intersect with immunomodulatory responses, immune tolerance, and regulatory immune processes. MNDs have an active and mechanistically grounded role in shaping the trajectory of AIDs. Evidence from experimental and clinical studies suggests that MNDs should be carefully considered, as they may amplify autoreactive immune responses and contribute to immune dysregulation, chronic inflammation, and disease progression.

Recent studies have also demonstrated clinical benefits associated with nutrition-based therapeutic approaches as an adjuvant to improve outcomes in different AID-related conditions, as well as in the potential reduction of pharmaceutical costs [94,95]. Based on these observations, micronutrient evaluation should be integrated into the clinical management of AIDs. Therefore, systematic screening protocols for micronutrient assessment should be included in the baseline evaluation of all newly diagnosed AID patients, with the aim of preventing and promptly treating potential deficiencies through monitored, personalized supplementation.

Future studies should aim to clarify which patient subgroups benefit most from targeted micronutrient interventions and nutritional optimization.

## Figures and Tables

**Table 1:** Current trials on nutritional interventions in patients with rheumatic diseases. ACPA, anti-cyclic citrullinated peptide antibody; AIDs, autoimmune diseases; CRD, chronic rheumatic disease; DAS28, Disease Activity Score in 28 Joints; ESSDAI, EULAR Sjögren’s Syndrome Disease Activity Index; ESSPRI, EULAR Sjögren’s Syndrome Patient-Reported Index; GSRS, Gastrointestinal Symptom Rating Scale; JIA, Juvenile Idiopathic Arthritis; NCWS, non-celiac wheat sensitivity; QoL, quality of life; RA, rheumatoid arthritis; SLE, systemic lupus erythematosus; SLEDAI, SLE Disease Activity; SjS, Sjögren’s Syndrome.

ID and Status	Title	Study Population	Design	Primary Outcomes
NCT06339957 Recruiting	Rheumatology Diet Study	Adult (18 years and older) patients with CRD	Observational Case-Only Cross-Sectional	How diet habits and activity affect AIDs
NCT05644795 Recruiting	Wheat-free Diet in the Treatment of SS	Adult (18 years to 64 years) patients with SjS	Randomized, Placebo-Controlled, Single Center	Self-perceived NCWS questionnaire; ESSPRI; ESSDAI, GSRS; extraintestinal symptoms rating scale.
NCT06474546 Recruiting	Feasibility of a Diet Intervention for Juvenile Arthritis (DIGEST-JA)	Patients aged 8–18 years with JIA	Single-Group Assignment	Feasibility, changes in QoL and disease activity, gut and systemic inflammation.
NCT06758817 Recruiting	The Triad of Nutrition, Intestinal Microbiota and RA (TASTY)	Adult (18 years and older) patients with RA	Randomized, Parallel Assignment	DAS28
NCT04748809 Recruiting	Effect of Anti-inflammatory Diet in RA	Adult (18 years and older) patients with RA	Randomized, Parallel Assignment	Clinical disease activity index
NCT07170033 Active, not recruiting	The Effect of the DASH Diet on Treatment Outcomes in Adults Diagnosed with RA	Adult (18 years to 64 years) patients with RA	Randomized, Controlled, Parallel Group	DAS28
NCT07183007 Not yet recruiting	Development of a Dietary Intervention Model Based on Genetic Data as an Implementation of a Healthy Lifestyle in the Management of SLE	Adult (18 years and older) patients with SLE	Randomized, Parallel Assignment	SLEDAI, QoL
NCT06776965 Recruiting	Ultra Processed Foods Consumption and Impact in Rheumatic Diseases. (NUTRIRIC)	Adult (18 years and older) patients with CRD	Prospective Cohort	Consumption of ultra-processed foods using a self-assessment questionnaire
NCT06874608 Recruiting	Evaluation of the Efficacy and Tolerability of an Exclusion Diet in Patients with JIA (JIA-ED)	Patients aged 6–18 years with JIA	Randomized, Parallel Assignment	Number of patients showing 20% improvement in juvenile arthritic disease activity score (JADAS)
NCT06659068 Active, not recruiting	Efficacy of Seluang Fish Oil (Rasbora Argyrotaenia) and Synbiotics Supplementation on CD4+ CD25+ Foxp3+ T-regulator Cells, IL17/IL-10 Ratio, and Disease Activity in SLE	Adult (18–55 years) patients with SLE	Single-Center Double-Blind Randomized Controlled	CD4+ CD25+ Foxp3+ regulatory T cells, IL-17/IL-10 ratio, SLEADAI-2K score
NCT06478290 Not yet recruiting	Pilot Assessment of the Safety of a Combination of Curcumin, Omega-3, and Vitamin D Supplements in ACPA+ Individuals (PASCOD3)	Adult (18–65 years) patients with ACPA+ based on serum testing	Single-Group Assignment	ACPA levels
NCT06654154 recruiting	Vitamin B12: a Biological Marker of Systemic Disease or Infection Flare-up in Patients Treated with Tocilizumab?	Adults (18 years and older) receiving intravenous or subcutaneous tocilizumab	Single-Group Assignment	Percentage change of vitamin B12 serum level between a remission state without infection and relapse or an infection

## Data Availability

No new data were created or analyzed in this study. Data sharing is not applicable to this article.
